# Deployment of Genetic and Genomic Tools Toward Gaining a Better Understanding of Rice-*Xanthomonas* *oryzae* pv. *oryzae* Interactions for Development of Durable Bacterial Blight Resistant Rice

**DOI:** 10.3389/fpls.2020.01152

**Published:** 2020-08-04

**Authors:** Anirudh Kumar, Rakesh Kumar, Debashree Sengupta, Subha Narayan Das, Manish K. Pandey, Abhishek Bohra, Naveen K. Sharma, Pragya Sinha, Hajira Sk, Irfan Ahmad Ghazi, Gouri Sankar Laha, Raman Meenakshi Sundaram

**Affiliations:** ^1^ Department of Botany, Indira Gandhi National Tribal University (IGNTU), Amarkantak, India; ^2^ Department of Life Science, Central University of Karnataka, Kalaburagi, India; ^3^ Department of Plant Sciences, School of Life Sciences, University of Hyderabad (UoH), Hyderabad, India; ^4^ Department of Biotechnology, ICAR-Indian Institute of Rice Research (IIRR), Hyderabad, India; ^5^ ICAR-Crop Improvement Division, Indian Institute of Pulses Research (IIPR), Kanpur, India

**Keywords:** rice, bacterial blight, resistance breeding, marker-assisted selection, genome editing, CRISPR/Cas, plant defense, transcriptome

## Abstract

Rice is the most important food crop worldwide and sustainable rice production is important for ensuring global food security. Biotic stresses limit rice production significantly and among them, bacterial blight (BB) disease caused by *Xanthomonas oryzae* pv. *oryzae* (*Xoo*) is very important. BB reduces rice yields severely in the highly productive irrigated and rainfed lowland ecosystems and in recent years; the disease is spreading fast to other rice growing ecosystems as well. Being a vascular pathogen, *Xoo* interferes with a range of physiological and biochemical exchange processes in rice. The response of rice to *Xoo* involves specific interactions between resistance (R) genes of rice and avirulence (*Avr*) genes of *Xoo*, covering most of the resistance genes except the recessive ones. The genetic basis of resistance to BB in rice has been studied intensively, and at least 44 genes conferring resistance to BB have been identified, and many resistant rice cultivars and hybrids have been developed and released worldwide. However, the existence and emergence of new virulent isolates of *Xoo* in the realm of a rapidly changing climate necessitates identification of novel broad-spectrum resistance genes and intensification of gene-deployment strategies. This review discusses about the origin and occurrence of BB in rice, interactions between *Xoo* and rice, the important roles of resistance genes in plant’s defense response, the contribution of rice resistance genes toward development of disease resistance varieties, identification and characterization of novel, and broad-spectrum BB resistance genes from wild species of *Oryza* and also presents a perspective on potential strategies to achieve the goal of sustainable disease management.

## Introduction

Rice (O*ryza sativa* L.) is an important staple food crop for more than 3.5 billion people across the world (Khush, 2005), provides 27 percent of the calories and 20 percent of protein required for the global population, and remains a major source of nutrition in developing and underdeveloped countries (FAO, 2004). Notwithstanding the progress witnessed in rice improvement over the last seven decades, the present rate of increase in rice yields are not adequate to keep pace with a rapidly growing population. The global demand of rice is estimated to rise by 26% in next 25 years, demanding an increase in its production from 676 million tones (mt) to 852 mt over the same period across the globe (Khush, 2013). Exacerbating the scenario, this production goal has to be achieved in the face of shrinking agricultural lands, dwindling water resources, declining soil productivity, and, most importantly, increasing cost of labor and other inputs. In parallel, we also need to improve rice production incrementally to combat and overcome the constantly evolving pathogen and pest populations and develop resilience in rice in the realm of rapidly changing climatic conditions.

Biotic stresses such as insect pests (brown plant hoppers, stem borers, etc.) and diseases such as bacterial blight caused by *Xanthomonas oryzae* pv. *oryzae*, rice blast caused by *Magnaporthe oryzae* and sheath blight caused by *Rhizoctonia solani* substantially reduce rice yields globally (Savary et al., 1998). The reduction in rice yield by bacterial blight (BB) is reported to be 50% (Khush et al., 1989), and during severe infection, it can reduce yield up to 81% (Srinivasan and Gnanamanickam, 2005), making it one of the most devastating diseases of rice (Ou, 1985). The conventional remedies recommended for managing BB disease, such as use of chemicals and antibiotics, biological control agents, and cultural practices, have limited utility and remain ineffective, especially when the disease occurs in epidemic proportion (Sundaram et al., 2008; Gnanamanickam, 2009).

Improving host-plant immunity has been considered as one of the best choices available for achieving economical and sustainable management of BB disease in a durable manner (Mundt, 2014; Pradhan et al., 2015). Understanding host resistance mechanisms and immunity against disease-causing pathogens like *Xoo* and their mutual interactions has been a topic of intense research in the past two decades. The evolving tools and techniques of plant molecular biology have been instrumental in getting vital insights into host-pathogen interactions and developing strategies for broad-spectrum, durable resistance. Chen and Ronald (2011) highlighted the nuances associated with innate immunity of rice against diverse pathogen elicitors. Typically, on encountering a biotic stress, the host plant reduces or enhances its susceptibility to a pathogen due to the existence of molecular cross talks between the pathogens themselves and between the pathogens and their host plant (Atkinson and Urwin, 2012). Interactions among pathways associated with response and tolerance/resistance to abiotic and biotic stresses have been established, and new insights have been gained on hormonal signaling pathways associated with antagonistic or synergistic interactions between biotic and abiotic stresses (Denancé et al., 2013; Jain et al., 2017). Therefore, understanding host-pathogen communication has been a longtime pursuit of plant biologists and plant pathologists.

Modern omics approaches like genomics, transcriptomics, proteomics, metabolomics, interactomics, etc. can be helpful in identification of the genes and their products, which are involved in pathogen perception by the host and also the response manifested by the host against the pathogen attack. Several resistance genes from different plant species have been identified, molecular mapped, cloned, and characterized (Sanseverino et al., 2010; Gururani et al., 2012). These genes have been assembled into five classes based on predicted protein domains (Gururani et al., 2012). *Hm1* gene of maize, which encodes a reductase, represents the first class. The HC toxin of *Cochliobolous carbonum* race 1 is inactivated by *Hm1* gene (Johal and Briggs, 1992). *Pto* gene belong to the second class, which encode membrane-associated serine-threonine kinase. It provides resistance to *Pseudomonas syringae* pv. *tomato.* The cytoplasmic receptor kinase protein represents the third class. It includes Rps2 and Rpm1, *N*, *L6*, *Prf*, and *Xa1* gene of *Arabidopsis*, tobacco, flax, tomato, and rice, respectively (Salmeron et al., 1996; Yoshimura et al., 1998). Tomato *Cf* gene represents the fourth class wherein *Cf* gene encodes LRR motifs in extracellular domain and a short C-terminal tail in the intracellular domain (Dixon et al., 1996). The rice *Xa21* represent the fifth class, which encodes a receptor kinase like protein, and it confers broad spectrum resistance to *Xoo* (Song et al., 1995).

In May 2014, the genome sequences of 3,000 strains of rice have been published by a joint effort of Chinese Academy of Agricultural Science (CAAS) and International Rice Research Institute, Philippines (Li et al., 2014). This project undoubtedly will provide the clear insights on utilizing host resistance genes belonging to one or more above mentioned classes of resistance genes for obtaining durable resistance against disease like BB (Li et al., 2014). In this article, we have reviewed the molecular mechanisms that are associated with interaction between rice and *Xoo*, pathways of resistance and susceptibility in rice, and the application of modern biotechnology approaches for breeding durable, broad-spectrum BB resistance rice varieties.

## Molecular Events Associated With Infection By Xoo And Resistance Against The Pathogen

### Plant-Pathogen Communication and Symptomatology

**Figure 1:**
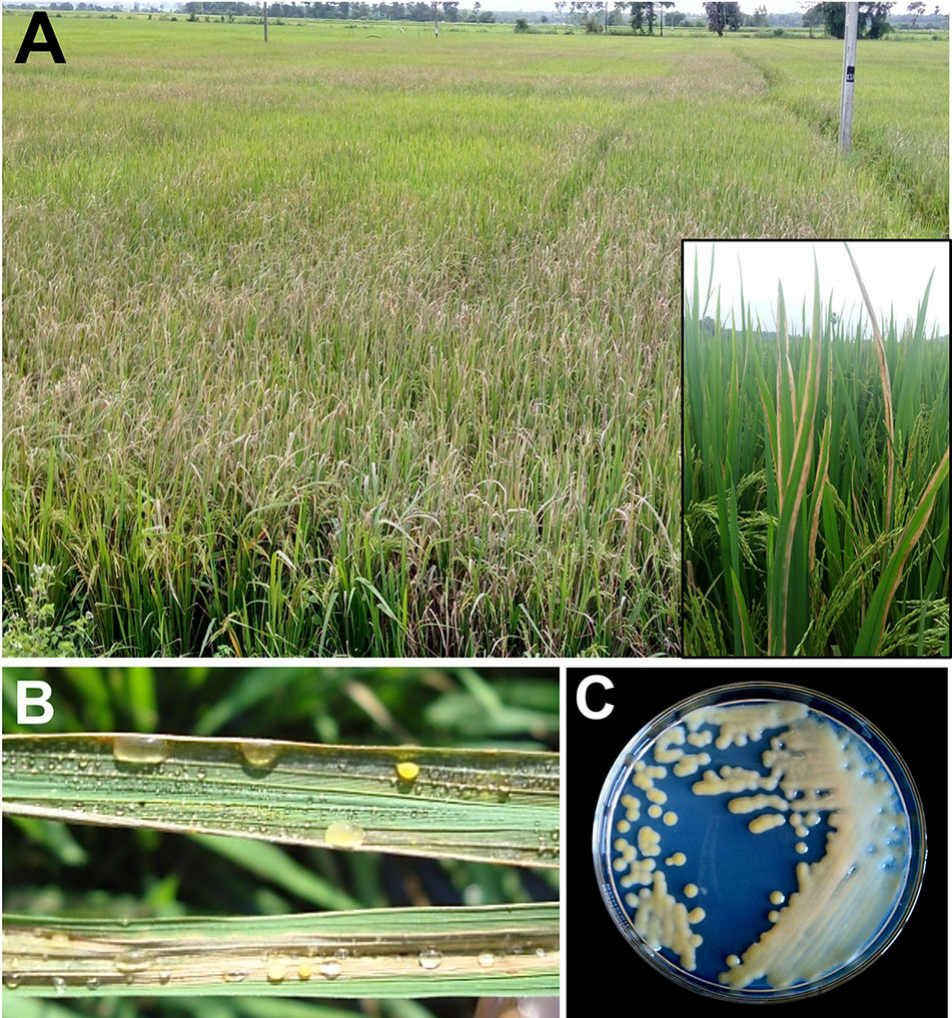
Bacterial blight of rice. **(A)** Closer view of infected plants; **(B)** bacterial ooze on infected leaf; **(C)** *Xanthomonas oryzae* pv. *oryzae* (*Xoo*) colonies on culture plate.

**Figure 2:**
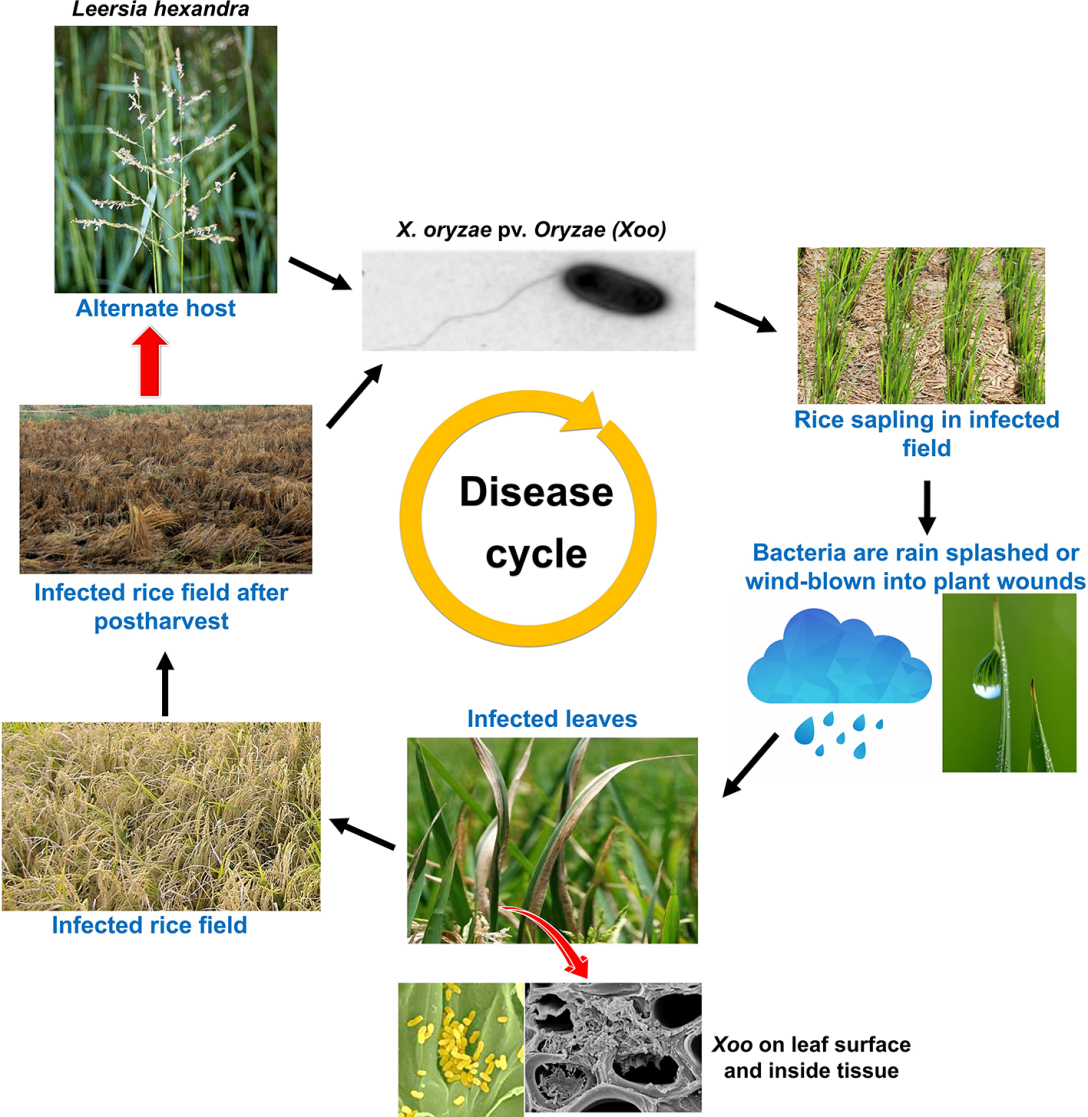
Disease life cycle of the rice bacterial blight caused by bacteria- *Xoo*, including the influence of disease secondary host plant on disease severity.

*Xanthomonas oryzae* pv. *oryzae* (*Xoo*) is a gram negative, non-spore forming, rod shaped bacterium, which is motile with a single polar flagellum. Individual cells show a range from around 0.7 to 2.0 µm in length and from 0.4 to 0.7 µm in width and require an optimal temperature between 25 and 30°C for their growth (Bradbury, 1984). Unlike mammals, plants have a complex cell wall and bacteria need to get through this barrier to gain access to nutrients. This is achieved by the bacteria through destruction of the cell wall barrier by means of secreting cell wall degrading enzymes (CDEs) such as lipase/esterase (*LipA*), cellulase (*ClsA*), cellobiosidase (*CbsA*), xylanase (*XynB*), etc., which is one of the most effective virulence strategy adapted by bacterial pathogens (Agrios, 1997; Rajeshwari et al., 2005; Jha et al., 2007; Malukani et al., 2020). However, receptors like WAKL21.2 predict the damage caused by *Xoo* CDEs and recruit the components of immunity (Malukani et al., 2020). *Xoo* gets into rice leaf tissues generally *via* wounds or natural openings such as hydathodes (Ou, 1985). Subsequently, it multiplies and flourishes in the intercellular spaces (apoplast) beneath the epithelial cells. Thereafter, it disseminates to other parts of plants through the xylem vessels (Noda and Kaku, 1999). After few days, xylem vessels are filled by bacteria and its exo-polysacchride (EPS). The bacterial with its exudates can be observed on the leaf surface (**Figures 1** and **2**), as they come out through hydathodes. This is considered as a clear-cut symptom of the disease and most importantly it serves as a source of secondary inoculum (Mew et al., 1993).

**Figure 3:**
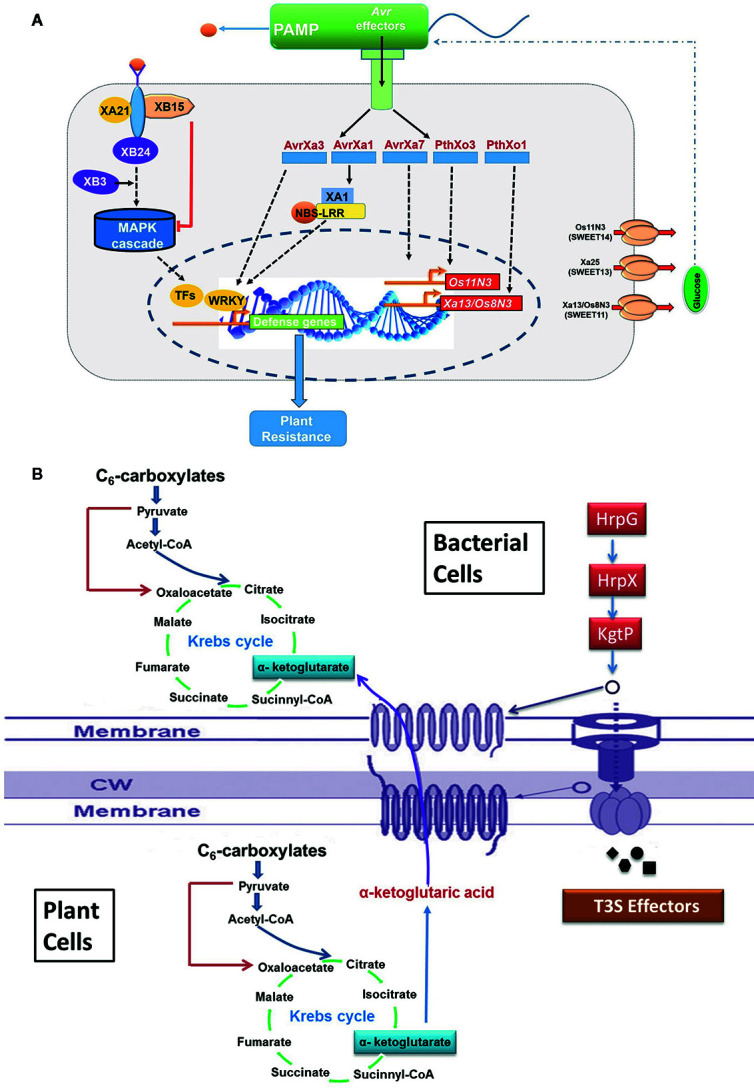
The schematic representation for the molecular signaling involved during host and pathogen interaction. **(A)** Hijacking host key genes (*SWEET11/13/14*) by pathogen; **(B)** utilization of host metabolic resources like Kreb’s intermediate by pathogen.

Many plant pathogenic bacteria including *Xoo* use type III secretion system to transport virulence proteins and enzymes to disrupt host signaling and hijacks host metabolism for their growth and development. The proteins secreted by type III secretion system are called effector protein, which includes transcription activator like (TAL) effector and non-TAL effector proteins (White et al., 2009; Scholze and Boch, 2011). TAL effector protein supports the proliferation of *Xoo* and establishment of infection in host plant by altering host transcription machinery through upregulation of selected host genes required for multiplication of the pathogen, whereas non-TAL effector protein promotes virulence through suppression of host innate immunity. Few TAL effectors and their cognate effector (E) genes, viz., *AvrXa10*/*Xa10*, *AvrXa23/Xa23*, and *AvrXa27*/*Xa27*, have been cloned from rice (Hopkins et al., 1992; Gu et al., 2005; Tian et al., 2014), and virulence function of others TAL effectors, *TalC*, *pthXo1*, *pthXo2*, *pthXo3*, *pthXo6*, and *pthXo7*, have been studied. The *PthXo1* TAL effector persuades the expression of host susceptibility gene *Os8N3* (*nodulin 3* gene family; renamed as *OsSWEET11*), which encodes a membrane protein associated with sugar transport (Yang B. et al., 2006). In the cultivar Nipponbare, it activates virulence by inducing *Os8N3*/*SWEET11* gene (**Figure 3A**) (Yang S. et al., 2006). It has been reported that the recessive *xa13* resistance allele arose due to mutation in the promoter region of *Os8N3*/*SWEET11* (Chu et al., 2006). Another host susceptibility gene, *SWEET14* is targeted by many TALEs (viz., *AvrXa7*, *PthXo3*, *TalC*, *and TalF*; Oliva et al., 2019) to trigger the release of sugar molecules in the apoplast required by the pathogen as nutrient source (Streubel et al., 2013). Mutation within effector binding element (EBE) of *AvrXa7* in the *Os11N3*/*SWEET14* promoter resulted in disease resistance against *Xoo* (Li T. et al., 2012). Similarly, deletion in the EBE of *Xa7* in wild rice confers broad spectrum resistance to BB (Hutin et al., 2015). Two other TAL effectors *PthXo6* and *PthXo7* promote the transcription of host genes *OsTFX1* and *OsTFIIAγ1*, respectively (Sugio et al., 2007). One more TAL effector gene *pthXo8* (homolog of *pthXo6*) has been found to be involved in manipulation of small RNA pathway of the host (Yang and White, 2004).

*Xoo* genome also encodes a type II secretion system. Proteins secreted by type II secretion system possess secretion signal at N terminal and are transported to periplasmic space (Voulhoux et al., 2001; Jha et al., 2005). Type II secreted (TIIS) proteins are mostly toxins and enzymes that targets diverse components of the host defense system. In addition, type II secretion system secretes variety of carbohydrate degrading enzymes like cellulases, pectate lyases, xylanases, and polygalacturonases (Cianciotto and White, 2017), thus weakening the cell wall. It has been noted that rice plants perceive type II protein and in response, hypersensitive reaction is inducted (Jha et al., 2005). Mutation in genes encoding type II secretion system diminishes the virulence of *Xoo* in the host, thus clearly demonstrating the importance of TIIS in plant pathogenesis (Cianciotto and White, 2017). *Xoo* targets and alters different host gene products and TAL effectors to amend the host physiology to have favorable effects on host susceptibility. The ability of plants to detect the adverse effects and speed of response against the pathogen determines the host fate. It has also been found that plants down-regulate the level of auxin in response to pathogen attack for enhancing disease resistance (Navarro et al., 2006). In addition, ABA suppresses the basal defense mechanism of rice against virulent *Xoo* strains and likely to function as virulence factor. An enhanced level of ABA is known to increase susceptibility of rice to *Xoo* by mediating the SA defense mechanism (Xu et al., 2013). *Xoo* also produce autoinducers (hormone like molecule) to detect the local population density (quorum sensing; QS) (Karatan and Watnick, 2009; Pradhan and Chatterjee, 2014). Based on the quorum sensing, bacteria regulate their gene expression pattern to effectively parasitize the plant cells (Karatan and Watnick, 2009). Various signaling molecules engage in QS including *N*-acylhomoserine lactones (AHLs), autoinducers-2 (AI-2), diffusible signal factors (DSFs), and oligopeptides (Deng et al., 2010). Further, the *Xoo* secretes large amount of extracellular polysaccharide (EPS; extra cellular polysaccharide high molecular-weight sugar molecules), which choke the xylem and cause typical wilting symptoms. EPS has an important role to play as it enhances pathogenicity by protecting the bacteria from antimicrobial compounds of the host plants (Leigh and Coplin, 1992; Dharmapuri and Sonti, 1999). All of the above mechanisms contribute together to promote pathogenesis (Leigh and Coplin, 1992). A comprehensive representation of the mode of *Xoo* infection in rice is illustrated in **Figures 1** and **2**.

### Host-Mediated Disease Resistance

The pathogen infects by evading or compromising the host defense responses. In doing so, the pathogen escapes the recognition by host receptors, mitigates or inhibits downstream signaling in the host, or takes over the host signaling mechanism to favor establishment of disease. To counter this, plants have also developed several receptors and sensors that interact with microbial components and nullify their effect. A unique strategy has been adopted to improve the immunity in crops by enhancing the recognition spectrum of the host plant’s own immune system (Lacombe et al., 2010). It involves the transfer of pathogen-associated molecular pattern (PAMP) like perception system across plant families and provides broad-spectrum disease resistance. Plant immunity has been categorized into two levels based on microbial component recognition. The first and second level of immunity is known as basal immunity [PAMPs-triggered immunity (PTI)] and gene-for-gene resistance [effector triggered immunity (ETI)], respectively (Jones and Dangl, 2006; Monaghan and Zipfel, 2012). Both PTI and ETI are mediated by receptor kinase proteins localized in plasma membrane and nucleotide binding (NB) leucine-rich repeat (LRR) proteins and other factors localized in cytoplasm respectively (Jones and Dangl, 2006; Macho and Zipfel, 2014). PTI provides quantitative resistance, and ETI provides qualitative resistance in plant pathogen interaction (Zhang and Wang, 2013). Rice-*Xoo* interaction is an exclusive example of qualitative resistance, i.e., major gene conferred resistance (Zhang and Wang, 2013). The major disease resistance genes of rice, which provide resistance to *Xoo*, fall under either ETI or PTI or may fall under an additional mechanism, different from ETI or PTI (Hu et al., 2017).

One important and unique feature that is typical of qualitative resistance of rice against *Xoo* is that one third of the major disease resistance genes are recessive genetically (Zhang and Wang, 2013; Hu et al., 2017). Out of the 44 known R-genes, at least 11 have been cloned and characterized (*Xa1*, *Xa3*/*Xa26*, *Xa4*, *xa5*, *Xa10*, *xa13*, *Xa21*, *Xa23*, *xa25*, *Xa27*, and *xa41*) (**Table 1**) (Tian et al., 2014; Wang et al., 2014a; Wang et al., 2014b; Cao et al., 2018). Of the remianing R genes, at least nine have been fine-mapped on different chromosome so far, viz., *Xa2*, *Xa4*, *Xa7*, *Xa22*, *Xa30*, *Xa33*, *Xa38*, *Xa39*, and *Xa40* (http://www.mshigen.nig.ac.jp/rice/oryzabase/gene/list). Interestingly, mutation in rice lines have also produced important R genes/alleles such as *Xa1*, *xa5*, *xa13*, *Xa23*, *xa25*, *Xa26/Xa3*, *Xa27*, and *xa41* (Nakai et al., 1988; Song et al., 1995; Yoshimura et al., 1998; Gao et al., 2002; Lee et al., 2003; Iyer et al., 2004; Sun et al., 2004; Gu et al., 2005; Chu et al., 2006; Liu et al., 2011; Wang et al., 2015; Hutin et al., 2015) (**Table 1**). Different R-genes encodes different types of proteins wherein *Xa1* encodes NB-LRR type protein (Yoshimura et al., 1998) and confers resistance to *Xoo* isolates by recognizing TALEs (Ji et al., 2016). *Xa21* and *Xa3*/*Xa26* encode plasma membrane localized LRR receptor like kinase proteins and confer race specific resistance to *Xoo* (Song et al., 1995; Li et al., 2012). *Xa4* encode cell wall associated protein kinase and boosts resistance to *Xoo* by strengthening the cell wall (Hu et al., 2017). The recessive gene *xa5* encodes gamma subunit of the basal transcription factor IIA 5 (TFIIAγ5) and is a substitution variant of a single amino acid V39E (Iyer and McCouch, 2004; Yuan et al., 2016). The non-variant version of the basal transcription factor is required for survival of *Xoo* in rice. The genes, *xa13*, *xa25*, and *xa41*, encode transmembrane proteins (Chu et al., 2006; Liu et al., 2011; Hutin et al., 2015; Cheng et al., 2017), which are basically sugar transporters, and the dominant alleles of these genes are specifically induced by TALEs produced by the pathogen for establishing infection. *Xa10* encodes an ER membrane protein, which elicits Ca^2+^ depletion in ER membrane inducing host cell death (Tian et al., 2014). *Xa23* is known to be an executor R gene that encodes a protein with 113 amino acid residues. The transcription of *Xa23* is triggered by *AvrXa23*, a TALE from *Xoo* (Wang et al., 2015). *Xa27* encodes apoplast protein, which triggers thickening of the secondary cell wall of the vascular bundle elements (Gu et al., 2004). Both dominant and recessive like *Xa1*, *Xa4*, *Xa21*, *xa5*, and *xa13* confer race specific resistance to *Xoo*, respectively, whereas the recessive alleles of genes such as *xa1*, *xa4*, and *xa21* and dominant alleles of *Xa5* and *Xa13* are susceptible to *Xoo* (Zhang and Wang, 2013). The cloned R genes, its cognate *Avr* genes, and the nature of the resistance of R genes have been summarized in **Table 2**.

**Table 1:** Genes conferring resistance to bacterial blight pathogens.

S.No.	Gene	Chr. No.	Source	Origin	Resistant to races	Reference
1	*Xa1*	4	Kogyoku	Japan	JR I	Yoshimura et al., 1998
2	*Xa2*	4	Tetep	Vietnam	JR II	He et al., 2006
3	*Xa3/* *Xa26*	11	Wase Aikoku3, Java-14, Chugoku-45, Cempocelek	Japan	PR 1,2,4,5 and All JR	Kaku and Ogawa, 2001; Sun et al., 2004
4	*Xa4*	11	TKM 6, IR 20, IR 22	India	PR 1, 4, 5, 7, 8 and 10	Wang et al., 2001
5	*xa5*	5	Aus boro lines (e.g. DZ192), DV85, DV86, DZ78	Bangladesh	PR 1, 2, 3, 5,7, 8, 9,10	Iyer and McCouch, 2004
6	*Xa6*	11	Zenith	USA	PR 1	Sidhu et al., 1978
7	*Xa7*	6	DV85, DV86, DZ78	Bangladesh	PR 1, 2, 3, 5, 7, 8,10	Lee and Khush, 2000; Porter et al., 2003
8	*xa8*	7	PI231128	USA	PR 5, 8	Sidhu et al., 1978; Singh et al., 2002
9	*xa9*	11	Khao Lay Nhay, Sateng	Laos	PRs	Singh et al., 1983; Ogawa, 1988
10	*Xa10*	11	Cas 209	Senegal	PR 2, 5, 7, JR	Yoshimura et al., 1983; Kurata and Yamazaki, 2006
11	*Xa11*	3	IR8, IR944	Philippines	JR IB, II, IIA, V	Mew, 1987
12	*Xa12*	4	Kogyoku,Tetep, Java-14	Japan	IR V	Mew, 1987
13	*xa13*	8	BJ1,Chinsurah Boro II	India	PR 6	Chu et al., 2006
14	*Xa14*	4	TN1	Taiwan	PR 5, 8	Oryzabase, 2006
15	*xa15*	ND	XM41 mutant	ND	JRs	Nakai et al., 1988; Gnanamanickam et al., 1999
16	*Xa16*	ND	Tetep	Vietnam	JI H8581and H8584	Oryzabase, 2006
17	*Xa17*	ND	Asominori	South Korea	JI H8513	Oryzabase, 2006
18	*Xa18*	ND	IR24, Toyonishiki, Miyang23	Philippines, Japan	BI-BM8417and BM8429	Liu et al., 2004; Oryzabase, 2006
19	*xa19*	ND	XM5 (mutant of IR24)	ND	PR 1, 2, 3, 4, 5 and 6	Lee et al., 2003; Oryzabase, 2006
20	*xa20*	ND	XM6 (mutant of IR24)	ND	JI	Taura et al., 1992
21	*Xa21*	11	*O. longistaminata*	Africa, Mali	PR 1, 2, 3, 4,5, 6, 7, 8 and 9	Song et al., 1995
22	*Xa22*	11	Zhachanglong	China	CRs (BSR)	Wang et al., 2003; Sun et al., 2004
23	*Xa23*	11	*O. rufipogon* (CBB23)	China/Cambodia	All PR, JR, CR, IR	Zhang et al., 1998; Zhang et al., 2001
24	*Xa24(t)*	2	DV85, DV86, Aus 295	Bangaladesh	PR 6, CRs	Khush and Angeles, 1999; Lee and Khush, 2000
25	*xa25/* *Xa25(t)*	12	Minghui 63, HX-3	China	PR 1,3 4, and to CR	Amante-Bordeos et al., 1992; Lee et al., 2003
26	*Xa26 (t)*	11	Minghui 63, Nep Bha Bong	China	PRs (BSR)	Yang et al., 2003; Sun et al., 2004
27	*Xa27*	6	*O. minuta* IRGC101141	Philippines	PR 2, 5	Gu et al., 2004; Gu et al., 2005 and Lee et al., 2003
28	*xa28(t)*	ND	Lota Sail	Bangladesh	PR 2, 5	Lee et al., 2003
29	*Xa29(t)*	1	*O. officinalis* (B5)	ND	CRs	Tan et al., 2004
30	*Xa30(t)*	11	*O. rufipogon* (Y238)	India	IR	Tan et al., 2004
31	*Xa31(t)*	4	*Zhachanglong*	China	CRs	Wang et al., 2009
32	*Xa32(t)*	11	O. australiensis	ND	PR PXO339	Chen et al., 2002
33	*xa33(t)/*	6	*Ba7, O. nivara*	Thailand	TRs	Korinsak et al., 2009
	*Xa33 (t)*					Natrajkumar et al., 2012
34	*xa34(t)/Xa34 (t)*	1	Pin Kaset, O. brachyantha	Sri Lanka	TRs	Korinsak et al., 2009; Ram et al., 2010
35	*Xa35(t)*	11	*O. minuta* (Acc.No, 101133)	Philippines	PRs	Guo et al., 2010
36	*Xa36(t)*	11	C4059	China	PR	Miao et al., 2010
37	*Xa37(t)*	ND	Unknown	ND	Unknown	
38	*Xa38*	ND	*O. nivara* IRGC81825	ND	IRs	Cheema et al., 2008 Kaur et al., 2006
39	*Xa39*	11	FF329	ND	CRs, PRs	Zhang et al., 2014
40	*Xa40 (t)*	11	IR65482-7-216-1-2		KRs	Kim et al., 2015
41	*Xa41(t)*	ND	Rice germplasm	ND	Various	Hutin et al., 2015
42	*xa42*	3	XM14 (mutant of IR24)	ND	JRs	Busungu et al., 2016
43	*Xa43(t)*	11	IR36 (P8)	ND	KRs	Kim and Reinke, 2019
44	*xa44 (t)*	11	IR73571-3B-11-3-K3 (P6)	ND	KRs	Kim, 2018

**Table 2:** List of cloned rice R genes, cognate *Xanthomonas oryzae* Avr genes, and nature of resistance of R genes (adapted from Jiang et al., 2020).

S.No.	Gene	Chromosome number	Encoded Protein	Cognate *Avr* gene	Encoded Protein	Resistance nature	Reference
1	*Xa1*	4	Nucleotide binding site–leucine rich repeat (*NBS*-*LRR*)	*PthXo1*/*Tal4*/*Tal9d*	ND	Race specific	Yoshimura et al., 1998; Ji et al., 2016
2	*Xa3/Xa26*	11	Leucine-rich repeat receptor-like protein kinase (LRR-RLK)	*AvrXa3*	ND	Broad spectrum	Sun et al., 2004; Li et al., 2004; Xiang et al., 2006
3	*Xa4*	11	Wall-associated kinase (WAK)	ND	ND	Race specific	Hu et al., 2017
4	*xa5*	5	TFIIAγ5 transcription factor	*Avrxa5*/*PthXo7*	TAL effector	Race specific	Jiang et al., 2006; Sugio et al., 2007; Zou et al., 2010
5	*Xa10*	11	Executor R protein/	*AvrXa10*	TAL effector	Broad spectrum	Tian et al., 2014
6	*xa13(OsSWEET11)*	8	SWEET-type protein/nodulin 3 family protein	*PthXo1*	TAL effector	Race specific	Chu et al., 2006; Yang B. et al., 2006; Yuan et al., 2009
7	*Xa21*	11	Leucine-rich repeat receptor-like protein kinase (LRR-RLK)	*RaxX*	ND	Broad spectrum	Song et al., 1995; Pruitt et al., 2015
8	*Xa23*	11	Executor R protein	*AvrXa23*	TAL effector	Broad spectrum	Wang et al., 2014b; Wang et al., 2015
9	*xa25(OsSWEET13)*	12	SWEET-type protein/nodulin 3 family protein	*PthXo2*	TAL effector	Race specific	Liu et al., 2011; Zhou et al., 2015
10	*Xa27*	6	Executor R protein	*AvrXa27*	TAL effector	Broad spectrum	Gu et al., 2005
11	*xa41(OsSWEET14)*	11	SWEET-type protein	*AvrXa7*/*PthXo3*/*TalC/Tal5*	TAL effector	Broad spectrum	Antony et al., 2010; Yu et al., 2011; Hutin et al., 2015; Hutin et al., 2015

TFIIA, transcription factor IIA; SWEET, sugar will eventually be exported transporter; TAL, transcription activator like; ND, not determined.

## Introgression of Novel BB Resistance Genes From Wild Relatives of Rice

The genus *Oryza* includes two cultivated species of rice, i.e., *O. sativa and O. glaberrima* (2n = 24, genome type AA) and 22 wild species (2n = 24, 48) containing an array of genome types, including those belonging to AA, BB, CC, BBCC, CCDD, EE, FF, GG, KKLL, and HHJJ (Goicoechea et al., 2010). The *Oryza*, belonging to wild species are considered as a repository of genetic diversity that can be an asset for crop improvement. Although wild relatives of rice are atrocious to the cultivated varieties in terms of many agronomic traits, they are potential genetic resource with tremendous genetic diversity (Ali et al., 2010). Presence of adaptive traits that are often lacking in cultivars renders them vulnerable to several biotic and abiotic stresses, while a majority of the wild rice can withstand harsh biotic and abiotic environmental conditions. Cultivated rice has been the source of many BB resistance genes, and introgression of these genes into elite varieties/hybrids has been done through conventional breeding or also through marker-assisted breeding (MAB). However, transfer of genes from wild species to cultivated types brings with it a set of challenges such as hybrid sterility, linkage drag, and incompatibility barriers. So far, a handful of BB resistance genes have been identified and introgressed from related wild species of *Oryza* into cultivars (Nino-Liu et al., 2006; Sanchez et al., 2013). With the advent of molecular and genomic tools such as trait-associated DNA markers, high-throughput marker-assisted genotyping rapid identification of BB resistant sources and the process of their introgression into elite cultivars can be accelerated tremendously.

The first BB resistance gene to be cloned and characterized in the rice was *Xa21*. It was originally identified and introgressed from an accession of *O. longistaminata* (AA genome), and the gene encodes a receptor kinase like protein and provides broad-spectrum resistance against BB (Song et al., 1995). *Xa21* has been transferred to several rice cultivars and hybrids through marker-assisted breeding (MAB) (Williams et al., 1996; Singh et al., 2001; Perez et al., 2008; Sundaram et al., 2008; Balachiranjeevi et al., 2018). Additional broad-spectrum BB R-gene *Xa23* (encoding executor R protein) was transferred into Asian cultivated rice from *O. rufipogon* (AA) (Zhang et al., 2001). Various wild species of *Oryza* pertaining to secondary gene pool have also assisted as a source of BB R-genes such as *Xa27* from *O. minuta* (BBCC) and *Xa29(t)* from *O. officinalis* (CC) (Amante-Bordeos et al., 1992; Tan et al., 2004; Gu et al., 2005). Other BB resistance genes isolated from wild relatives and characterized with the help of molecular markers and genomic tools include *Xa10* (Gu et al., 2008; Tian et al., 2014), *Xa30* (*O*. *rufipogon*) (Jin et al., 2007), *Xa32* (*O*. *australiensis*) (Zheng et al., 2009), and *xa*32 (*O*. *meyeriana*) (Ruan et al., 2008), *Xa32t* (*O. australiensis*), *Xa33* (*O. nivara*) (Natrajkumar et al., 2012), *Xa35t* (*O. minuta*), and *Xa38* (*O. nivara*) (Cheema et al., 2008). A novel locus on chromosome 12 of *O. latifolia* (wild allotetraploid rice species) was identified recently, and it confer race specific resistance of *Xoo* strain PXO339 (Angeles-Shim et al., 2020). Based on these developments, it can be inferred that wild relatives of *Oryza* are expected to contribute significantly in developing durable BB resistant rice varieties. Whole genome sequencing of wild rice will expedite identification of resistance genes from the wild relatives of rice and may offer insights about the the pathways associated with the evolution of different resistance genes.

In additional to wild rice resources, it is generally accepted that durable and broad-spectrum resistance against plant dieseases can be enhanced by deployment of quantitative trait loci (QTLs) along with major genes so that both vertical and horizontal resistance can be achieved. In the recent past, a few studies highlight the role of QTLs with respect to resistance/tolerance against BB of rice. Even though few QTLs associated with tolerance/resistance to BB have been reported earlier, most of these QTLs mapped closely to already identify major resistance genes (Li et al., 1999). Five major QTLs were maped on various chromosomes for African resistant *Xoo* strains *Xoo*. Various loci on different chromosomes such as 1, 7, 9, 10, and 11 explained as much as 13%, 37%, 13%, 11% and 15% of phenotypic variation in terms of resistance, respectively (Djedatin et al., 2016). A major *qBBS11* was identified by composite interval mapping of MAGIC population derived from Japonica, and it explained 31.25% of the phenotypic variation (Kim and Reinke, 2019), and this QTL was later renamed as *Xa43(t)*. On closer examination, *xa34(t)* has been identified to co-localize along with *qABB*-1 on rice chromosome 1, which is a resistance QTL induced by the African *Xoo* strain (Chen et al., 2011).

## Analyzing Resistance Gene Analogues (RGAs) As A Novel Tool To Identify Blight Resistance Gene

Resistance genes analogs (RGAs) are a large class of disease resistance associated genes and they can be categorized into two major groups, namely NBS-LRR and transmembrane LRR (TM-LRR) (Hammond-Kosack and Jones, 1997; Sekhwal et al., 2015). Others include pentatricopeptide repeats (PPRs) and apoplastic peroxidases. NBS-LRR class of RGAs targets effector protein of pathogen, thus mediate effector triggered immunity (ETI) in host cell, whereas TM-LRR class of RGAs mediates PTI (Chisholm et al., 2006). NBS-LRR represents the most abundant and best-known family of RGAs contributing to disease resistance in plants (Porter et al., 2009). Analysis of whole genome sequences of japonica Nipponbare and indica 93-11 suggested presence of RGAs in pseudogenes, with 347 RGAs in Nipponbare and 345 in 93-11 as pseudogenes (Luo et al., 2012). Interestingly, most of the identified pseudogenes have strong identity with one or the other NBS protein (Liu et al., 2011). Further, many studies have shown that the RGAs are randomly distributed on chromosome either in large or small clusters (Ghazi et al., 2009), for example, 50% NBS and 74.3% NBS-LRR class of RGAs were found to be clustered in rice (Yang S. et al., 2006). The distribution of RGAs in clustered manner potentially functions like reservoir of genetic variation, which may be responsible for bringing the evolution of new *R* genes (Michelmore and Meyers, 1998; Young, 2000; Zhou et al., 2007). On the long are of chromosome 11, cluster of six *Xa21* like RGAs was reported. The NBS-LRR containing genes cluster was also predicted at 0.6 Mb away from *Xa21*, which indicates the existance of extra NBS-LRR–type genes for activation and expression of the *Xa21* gene (Ghazi et al., 2009). Therefore, from the application point of view, RGAs provide enormous opportunities as they can be used as candidates’ genes for R-gene mapping and cloning, co-localization of QTLs, SNP marker development, and for resistance breeding (Liu et al., 2007; Huang et al., 2012).

## Modern Approaches for Development of BB Resistant Rice

### Molecular Breeding for BB Resistance in Rice

Efforts have been made to improve resistance of rice against BB through conventional and modern breeding techniques. This is achieved through standard crossing and/or backcrossing an elite rice variety/hybrid with the genotype carrying the resistance gene to BB. The practice not only reduces the use of chemical pesticides but also offers a sustainable way for management of this disease. Presence of several virulent bacterial strains in the rice growing areas throughout the world necesssitates cultivation of such rice varieties, which are endowed with multiple resistance genes as gene-pyramids. To date, identification of 44 R genes conferring resistance to diverse *Xoo* races has been completed (Busungu et al., 2016; Neelam et al., 2019); majority of these identified genes come from *O. sativa* ssp. *indica* or *japonica*. As mentioned earlier, a set of resistance genes have been also identified from wild species of rice such as *O. longistaminata*, *O. rufipogon*, *O. minuta*, and *O. officinalis* (Bhasin et al., 2012; Kumar et al., 2012; Wang et al., 2014a; Zhang et al., 2014; Kim et al., 2015) (**Table 1**).

It is pertinent to note that 14 R-genes (*xa5*, *xa8*, *xa13*, *xa15*, *xa19*, *xa20*, *xa24*, *xa25*, *xa26b*, *xa28*, *xa31*, *xa32*, *xa33*, and *xa34*) out of 44 known R-genes are recessive, while the others are dominant in their inheritance, and *Xa27* has shown both dominant and semi-dominant inheritance in different genetic backgrounds (Gu et al., 2004; Chen et al., 2011; Kim et al., 2015; Cao et al., 2018) (**Table 1**). The incorporation of several resistance (R) genes has been facilitated through marker**-**assisted backcrossing *(*MABC) or conventional backcross breeding, and resistance breeding has played a significant role in defending rice from the attack by the pathogen (Sundaram et al., 2008; Sundaram et al., 2009; Perumalsamy et al., 2010; Hari et al., 2011; Hari et al., 2013; Pandey et al., 2013; Kim et al., 2015; Balachiranjeevi et al., 2015; Abhilash et al., 2016; Abhilash et al., 2017). A rice derived BB resistance gene *Xa38* was incorporated into a BB susceptible rice variety PB1121 and APMS 6B (a rice maintainer line), either singly or in combination with other BB resistance genes, using a modified MABC approach, and improved lines showed broad spectrum of resistance against different *Xoo* races (Ellur et al., 2016; Yugander et al., 2019). Rice varieties carrying single resistance genes (e.g., *Xa4*) are not recommended for long term cultivation, as acute selection pressure on the pathogen results in rapid evolution of compatibility between *Xoo* and rice (Mew et al., 1992). Harnessing broad-spectrum resistance through pyramiding multiple resistance genes can be helpful to avoid such breakdowns. The probability of breakdown in cases of resistance conferred by two or more genes in a single genotype is much lower than that of a single gene controlling resistance (Mundt, 1990). For instance, R-genes in combination (*Xa4/xa5* and *xa5/Xa21*) offer higher level of resistance compared to both parental level and single gene (Sattari et al., 2014; Pradhan et al., 2016). Huang et al. (1997) developed four-gene pyramid lines comprising *Xa4*, *xa5*, *xa13*, and *Xa21* genes in IR24 cultivar genetic background through MABC, and the gene-pyramid lines showed broad spectrum disease resistance. Several research groups have performed BB gene pyramiding in rice using MABC techniques, e.g., marker-assisted introgression of *xa5*, *xa13*, and *Xa21* in the genetic background of PR106, Samba Mahsuri, Triguna, and Jalmagna (Singh et al., 2001; Sundaram et al., 2008; Sundaram et al., 2009; Pradhan et al., 2015). Similarly, *xa13* and *Xa21*genes pyramiding was carried out in the genetic background of Pusa Basmati 1 (Joseph et al., 2004). **Table 3** offers a comprehensive list of BB resistance genes that have been deployed or in the process of deployment in rice through MAB.

**Table 3:** Cultivars improved for bacterial blight resistance through breeding/marker-assisted breeding.

S.No.	Resistance genes	Variety/Parental line	References
1.	*Xa21*	Swarna	Reddy et al., 1997
2.	*Xa21*	Minghui63	Chen and Zhang, 2000
3.	*Xa21*	KMR3R	Hari et al., 2011
4.	*Xa33*	Samba Mahsuri	Natrajkumar et al., 2012
5.	*Xa4, xa5*	Angke	Sattari et al., 2014
6.	*xa5*, *xa13*	Triguna	Sundaram et al., 2009
7.	*xa13*, *Xa21*	Pusa Basmati1	Joseph et al., 2004
8.	*Xa7, Xa21*	Minghui63	Zhang et al., 2006
9.	*Xa21, xa13*	Pusa6B, PRR78	Basavaraj et al., 2010
10.	*Xa21, xa13*	Taraori Basmati, Basmati386	Pandey et al., 2013
11.	Xa21, xa13, sd-1	Type 3 Basmati	Rajpurohit et al., 2010
12.	*Xa4*, *xa-5*, *Xa21*	Mangeubyeo	Suh et al., 2013
13.	*Xa4*, *Xa7*, *Xa21*	TGMS1	Perez et al., 2008
14.	*xa5*, *xa13*, *Xa21*	Samba Mahsuri	Sundaram et al., 2008
15.	*xa5*, *xa13*, *Xa21*	Jalmagna	Pradhan et al., 2015
16.	*Xa5*, *xa13*, *Xa21*	ADT43, ASD16	Perumalsamy et al., 2010
17.	*xa5*, *xa13*, *Xa21*	IR65598-112	Sanchez et al., 2000
18.	*xa5*, *xa13*, *Xa21*	PR106	Singh et al., 2001
19.	*xa5*, *xa13*, *Xa21*	IR64	Davierwala et al., 2001
20.	*Xa4*, *xa5*, *Xa7*	IR64	Leung et al., 2004
21.	*Xa21, xa13, Xa38*	Pusa Basmati 121	Ellur et al., 2016
22.	*Xa4*, *xa5, xa13, Xa21*	Lalat, Tapaswini	Dokku et al., 2013
23.	*Xa4*, *xa5*, *xa13*, *Xa21*	IR24	Huang et al., 1997
24.	*Xa4*, *xa5*, *Xa13*, *Xa21*	Mahsuri	Guvvala et al., 2013
25.	*Xa4*, *xa5*, *xa13*, *Xa21*	Swarna, IR64	AICRIP, 2008
26.	*Xa7, Xa21, Xa22, Xa23*	Huahui1035	Huang et al., 2012

### Development of Transgenic Rice Resistant to BB

A potential strategy to control BB disease is the genetic transformation of elite cultivars using cloned resistance genes. Compared to conventional breeding, it is less time-consuming and avoids the problem of linkage drag. The first transgenic line containing *Xa21*, T-309, was developed in *japonica* rice by Wang et al. (1996). Later, *Xa21* was introduced into several varieties such as IR 72, MH 63, and IR 51500 (Datta et al., 2002; Tu et al., 1998; Tu et al., 2000). Similarly, an elite restorer line genetically transformed with *Xa21* has shown marked level of resistance to BB while retaining its original traits (Zhai et al., 2004). Field trials of *Xa21* transgenic rice conducted in India, Philippines, and China led to the identification of BB-resistant lines in transgenic IR 72 (Datta, 2004). Transgenic lines carrying one or the other *Xa* genes were developed for functional characterization of the gene; however, none of these could be commercialized due to regulatory and policy bottlenecks. Hence, conventional breeding combined with marker-assisted breeding or genomics assisted breeding is the preferred strategy for developing resistant lines/varieties to control BB disease.

### Mining of Novel Alleles of BB Resistance Genes

Mining superior alleles from different gene pools of any crop provides opportunity to access novel and effective alleles for biotic and abiotic stresses, which can be deployed in plant breeding for cultivar development (Ramkumar et al., 2010). To identify novel or superior allele of the known gene among the population, PCR-based approach is widely used. In this method, PCR amplification of homologs from different wild and cultivated germplasm is performed and analyze the PCR amplicon. This method is known as allele mining, and it has been widely studied for BB resistance genes such as *Xa7* (Utami et al., 2013), *Xa27* (Bimolata et al., 2013), *Xa26*, *Xa21*, *and xa5* (Bimolata et al., 2015), etc. This method will also reveal the degree of conservation among genes and other regulatory regions across the species. As mentioned earlier, fast adaptation of the pathogen races causes the failure of disease resistance in varieties containing single resistance genes and even two genes. Therefore, discovery of novel sources of resistance becomes crucial to match the plasticity of rapidly evolving pathogenic *Xoo* strains (Barry et al., 2007). In this context, surveying the genic/allelic variation available in landraces (Borba et al., 2009), traditional varieties, and wild relatives (Barbier, 1989) of rice will contribute to obtain effective and durable BB resistant varieties. One such example is *Xa7*, which has been used to develop BB resistance rice varieties (Perez et al., 2008; Utami et al., 2010). Allele mining approach was applied in the local rice accessions of Indonesia (Parekaligolara) to isolate resistant alleles of *Xa7* (Utami et al., 2013). Subsequently, these accessions containing variant alleles with respect to *Xa7* have been used as for developing new BB resistant rice lines (Utami et al., 2013). Analysis of variation in amino acid residues between the resistant and susceptible lines has revealed co-linear non-synonymous substitutions of lysine-cysteine-valine to serine-serine-threonine, respectively. Another BB resistance gene, *Xa27* provides resistance to rice against only those strains of *Xoo* harboring the avirulence gene *avrXa27*. Both dominant and recessive alleles of *Xa27* gene code for same protein without any changes in the protein sequence, but polymorphism exists in their promoter sequences. Deletion of three nucleotides AGA at 51^st^ position in the promoter of recessive allele (i.e., non-functional allele) of the gene has been reported (Gu et al., 2005). Twenty-seven alleles of *Xa27* gene have been identified in *O. nivara* and *O. sativa* at both promoter as wel as 5´UTR region. Such nucleotide diversity analysis will certainly help in diversification of gene function and enhancing the intensity of BB resistance (Bimolata et al., 2013). The nucleotide diversity analysis of naturally occurring BB resistance genes (*Xa21*, *Xa26*, and *xa5*) alleles was carried out in diverse cultivars of *Oryza* species and their wild relatives (Bimolata et al., 2013). The highest singleton variable sites (SVS) and nucleotide diversity were reported in *Xa26*, whereas maximum frequency of single nucleotide polymorphisms (SNPs) was observed in *Xa21*. Many substitutions and InDels resulted in nucleotide and amino acid polymorphism at *Xa21* and *Xa26* loci, which also have pathogen recognition LRR domain, and finally resulted in non-functional gene. Transition bias was reported in all the three alleles of *Xa21*, *Xa26*, and *xa5*, where G to A transition was favored more (Bimolata et al., 2015). Functional characterization of the new alleles will help in deciphering their actual roles in resistance against the pathogen. Alternative promising approach is RNA interference (RNAi) technique, which is used to silence molecules involved in regulating resistace genes negatively (Gust et al., 2010; Li C. et al., 2012). The final outcome results as a stronger and durable defense response, which leads to reduce disease manifestation and progression.

### Mutagenesis and TILLING

Targeting induced local lesions in genomes (TILLING) is a reverse genetic non-transgenic technique that is exploited to detect induced mutations in the target genes for the improvement of both plant and animal species (Barkley and Wang, 2008). Recently, with the aim to develop disease resistance varieties and creating useful genetic variation for multiple traits, TILLING population have been generated in many economically important plants like wheat (Fitzgerald et al., 2010), barley (Talamè et al., 2008), tomato (Piron et al., 2010), sunflower (Sabetta et al., 2011), and melon (González et al., 2011) by employing either chemical or physical mutagens. In rice, Till and colleagues (2007) could successfully generate a high-density TILLING population (1 mutation/250–300 kb) in Nipponbare variety of rice. Wu et al. (2005) demonstrated the efficacy of TILLING approach for identifying mutant rice lines with enhanced resistance against BB, rice blast and tungro virus, with a frequency ranging from 0.01 to 0.1%. They generated 60,000 IR64 TILLING mutant lines by using chemical and physical mutagens, out of which 38,000 unique lines were advanced to M4 generation for forward and reverse TILLING. In rice, the technique has been employed to isolate various rice mutants by targeting important agro-economical genes such as *OsBZIP* for rice blast resistance (Till et al., 2007); *OsTPS1*c *OsDREB*, *OsSNAC1*, *OsAKT1*, *OsHKT6*, *OsNSCC2*, *OsHAK11*, *OsSOS1*, *OsAHP1*, and *OsPLA1* for abiotic resistance (Till et al., 2007; Suzuki et al., 2008; Casella et al., 2013; Hwang et al., 2016); *OsSD1* for regulating plant height (Casella et al., 2013); *OsHd1* and *OsSAD* flowering (Suzuki et al., 2008; Casella et al., 2013); *OsACOS12* for fertility (Li et al., 2016); and *OsBADH2* for aroma (Casella et al., 2013).

TILLING population can also be used for forward genetic studies. Screening of 60,000 IR64 (rice *indica* cultivar) mutants led to identification of several loss or gain of resistance mutants of showing resistance or enhanced susceptibility to BB, blast, and tungro diseases (Wu et al., 2005). EMS induced mutant in the genetic background of Nagina 22 rice variety has been developed and utilized for various genetic studies in India (Sevanthi et al., 2018). It may be worthwhile to screen the rice lines for resistance against BB and other biotic stress and identify novel genetic variations. In addition to the above, we have generated a large sized EMS mutagenized population of Samba Mahsuri and screened them for resistance against bacterial blight. Preliminary results show that few of the mutant lines of Samba Mahsuri display enhanced resistance (Ershad Gopi et al., 2017). These mutagenized populations can be shared with researchers or breeders for rapid screening for a range of phenotypes, and the TILLING populations can serve as a public resource for the research community. For instance, IRRI, Philippines has distributed around 15,000 mutant lines of IR64 to researchers worldwide for detection of novel phenotypes, including sensitivity to plant hormones, phytic acid abundance, response to salinity and drought, and non-host resistance (Wu et al., 2005). A public TILLING support tool (http://tilling.ucdavis.edu/index.php/RiceTilling) and rice mutant database (IRIS; http://www.iris.irri.org) have been established in rice.

### Sequencing Based High-Throughput Mutation Detection Systems

In rice, NGS based TILLING protocols are well documented (Burkart-Waco et al., 2016) and it is a matter of time before the strategy is widely adopted to identify genes associated with resistance/susceptibility against bacterial blight pathogen in rice. Unlike NGS, where one can obtain the exact information of nucleotide base change and its position caused due to mutagen, high-resolution melting (HRM) identifies the mutation based on the differences in the melting curve of fragments of mutant and wild type. HRM technique is particularly important for analyzing the target genes that consist of multiple exons of smaller lengths. Though NGS and HRM techniques are still costlier, their capacity to generate results within a very short span of time is indeed encouraging. NGS- and HRM-based TILLING or Eco-TILLING strategy could uncover a new set of genes controlling BB resistance, and hence expedite the progress of developing new rice cultivars with greater level of resilience. In this context, it is worthwhile to note that International Rice Research Institute (IRRI), Philippiens along with other collaborators have sequenced ~ 3000 rice accessions and the rice lines can be mined for novel alleles of major, cloned and well characterized genes conferring BB resistance.

### Genome Editing

Breeding traits of agronomic significance largely relies upon existing allelic variation and involves repeated cycles of crossing and selection to obtain a crop genotype with desired level of improvements, which may consume considerable time and efforts (Sundaram et al., 2014). These limitations can be overcome by using emerging genome editing technologies. Genome editing based on artificial nucleases is a transformative technology that has the ability to modify plant genomes in an accurate and expectable manner. So far, four sequence-specific nucleases, i.e., transcription activator-like effector nucleases (TALENs) (Christian et al., 2010), zinc finger nucleases (ZFNs) (Kim et al., 1996), meganucleases (Smith et al., 2006) and the CRISPR/Cas (CRISPR-associated) nucleases, have been successfully used in genome editing in many crop plants (Bortesi and Fischer, 2015). In principle, all these technologies can be used for modifying plant traits such as disease resistance. For example, TALEN technology was applied successfully to mutate a BB susceptible gene, *Os11N3/OsSWEET14* promoter in rice. The inability of the effector to bind to the promoter of *OsSWEET14*, ultimately resulted in BB resistance (Li et al., 2012). The *Os11N3* is a sucrose-efflux transporter family gene (i.e., *SWEET* gene) whose expression gets activated by *Xoo* effectors for the pathogen’s nutritional needs. By editing the effector-binding element (EBE) of *Os11N3*, the virulence function of effectors produced by *Xoo* was abolished, leading to improved BB resistance (Li et al., 2013). CRISPR/Cas9 mutagenesis of another susceptibility gene encoding sucrose transporter *OsSWEET13* was performed to achieve BB resistance. *Xoo* effector/TALE *PthXo2* induces the expression of *OsSWEET13* in host, which subsequently resulted in establishment of *Xoo* and host susceptibility (Zhou et al., 2015; Borrelli et al., 2018). The expression of *OsSWEET13* gene has been evidenced to be activated by binding of the TALE, *PthXo2* to EBE of its promoter sequence (Xu et al., 2019). Genome edited lines showed significantly higher level of resistance to pathogen strains possessing the TALE. This study exemplified the fact that the technology can be applied to elite rice varieties to edit multiple genes simultaneously or sequentially to provide stronger and durable resistance against majority of BB strains. Furthermore, better understanding of SWEET genes and CRISPR/Cas9-mediated genome editing tool has helped in producing broad spectrum resistance in Kitaake, IR64 and Ciherang-Sub1 rice varieties through genome editing (Oliva et al., 2019; Xu et al., 2019). Recently, CRISPR/Cas9-mediated genome editing in the promoter region EBEs of *OsSWEET14* gene has been demonstrated to confer resistance in Super Basmati rice lines against *Xoo* strain carrying *AvrXa7* TALE (Zafar et al., 2020).

Among the currently available nuclease-based genome editing tools, CRISPR/Cas system is the latest and more popular technology, which relies on RNA-guided engineered nucleases (Jinek et al., 2012). CRISPR/Cas method employed for genome editing consists of a Cas9 endonuclease targeting a specific sequence of the genome defined by a single guide RNA. The CRISPR/Cas technology is simple and efficient, more importantly, has the ability to cleave even methylated DNA (Hsu et al., 2013), and has less or no off-target mutations (Shen et al., 2014). Therefore, CRISPR/Cas system is more versatile for editing plant genomes with highly methylated CpG sites (Miao et al., 2013) and will the most desirable system for editing sequences of rice susceptibility genes like *OsSWEET11*, *OsSWEET13*, *OsSWEET14*, etc. Broad spectrum resistance against *Xoo* was reported in the transgenic rice lines, in which promoter region EBEs sequence of *OsSWEET11*, *OsSWEET13*, and *OsSWEET14* were edited through CRISPR/Cas9 technology (Xu et al., 2019). Transgenic wheat plants with mutated mildew resistance locus (*MLO*) obtained by CRISPR/Cas9, and TALEN technologies showed improved resistance to powdery mildew (Wang et al., 2014). With constant refinements in the technical aspects with respect to specific targeting of the desired gene sequence with precision, genome editing will certainly be a method of choice for developing disease resistant varieties in rice.

### Genomic Selection (GS)

Current crop breeding approach largely depends on the robust phenotyping and deployment of genetic markers. Major limitation of marker-assisted selection in plant breeding is use of biparental mapping population for QTL prediction and its applicability with traits associated with major effect genes but may work for polygenic traits that are controlled by many genes of small-effect, and in general, such traits are crucial for the improvement of new crop varieties (Heffner et al., 2009; Crossa et al., 2017). To improve the crop selection procedure, breeders have now adopting a newer model—a black box approach **‘**Genomic Selection Model**’** that does not solely depend on the prior knowledge about the effect or function of individual genetic markers. In fact, GS involves huge set of phenotyping surveillance along with all molecular marker information which avoid biased marker effect estimates; thereby, it can capture more of the variations that appears due to small-effect QTLs (Heffner et al., 2009). Further, GS is an improved form of MAS which concurrently estimates a genomic estimated breeding value for all locus, haplotype, or marker across the entire genome of each genotype. Therefore, genomic selection offers opportunity to increase grain yield and quality by rapid selection of superior genotypes and accelerating breeding cycle in less time. In recent years, GS and genomic-enabled prediction (GP) have been studied in rice for enhancing grain yield by analyzing the genetics and the statistical complexity, which includes environment interaction with genotype that control trait phenotype (Spindel et al., 2015; Xu et al., 2018). The genomic prediction model upon cross validating 363 elite breeding lines from IRRI predicted with an accuracy that ranged from 0.31 to 0.34 for grain yield and 0.63 for flowering time (Spindel et al., 2015). Similarly, Xu et al. (2018) used 575 rice hybrids as a training population, and 362,760 potential hybrids were used to predict agronomic traits such as branch number (primary and secondary) and per panicle grain number and primary branch with accuracy of 36.12, 61.80, and 75.83%, respectively. It can be expected that, in coming years, rice breeding will deploy GS model and GP prediction to rapidly identify novel loci associated with BB resistance and other productivity related traits and quickly use them in breeding programmes.

## Molecular Omics Approaches for Studying Rice-Xoo Interaction

### Transcriptome Analysis

In the last 15 years, more than 70 key genes providing resistance to different plant pathogen have been identified, cloned and characterized from different plant species (Sharma et al., 2009; Sanseverino et al., 2010). Pathogen incursion can alter the transcript levels of various host plant genes (Iqbal et al., 2005), and several techniques have been developed in recent years to study the differential expression pattern of host genes associated with response to pathogen attack and resistance against it. Some of these expression profiling techniques are cDNA microarray (Schena et al., 1995), cDNA- amplified fragment length polymorphisms (AFLP) (Vuylsteke et al., 2007), suppression subtractive hybridization (Diatchenko et al., 1996), serial analysis of gene expression (SAGE) (Velculescu et al., 1995; Mardis, 2008), digital gene expression (DGE) (Audic and Claverie, 1997), qPCR (Mortazavi et al., 2008), etc. Transcriptome analysis helps elucidate key genes and pathways participating in defense signaling during plant pathogen interaction (Glazebrook, 2001).

cDNA microarray study of a transgenic rice (TP309-Xa21)-*Xoo* (P6 and K1avirulent and virulent strain respectively) interaction observed 454 and 498 DEGs in the incompatible and compatible interactions, respectively, of which co-regulated genes were 237 (Li Q. et al., 2006). Ethylene receptor-like protein, ethylene-insensitive protein, protein phosphatase, and ADH were upregulated only in rice-*Xoo* incompatible interaction (Li Q. et al., 2006). A genome-wide identification of defense response genes was performed in *xa13* gene mediated resistance plants wherein 702 unique expressed sequences triggered by *xa13* were identified (Chu et al., 2004). Sequence analysis showed induction of homologs to putative R-genes encoding NBS-LRR and XA21 like protein. There was also induction of gene homologs related to host-pathogen interaction reported in other plant species, such as PR proteins, peroxidases, WRKY transcription factors, GST, RNA helicases, ubiquitins, catalase, ankyrin-like protein, and cytochrome P450 (Chu et al., 2004). Microarray analysis after infection by BXO43 strain of *Xoo* in the resistant variety of rice, Ajaya (IET 8585), and susceptible variety, IR24, has revealed the differential expression of 274 genes between and susceptible genotypes (Grewal et al., 2012). Out of 274 genes, 152 and 122 were reported to be up- and down-regulated, respectively, in IET8585 compared to IR24. Some of the major up-regulated transcripts include chitinase precursor, WRKY69, Hin 1, DREB1B, NB-ARC domain containing protein, glutathione S-transferase (GST), cytochrome P450, harpin-induced protein, lipoxygenase, and flavanoid 3-monooxygenase (Grewal et al., 2012). It was interesting to note that several defense related signaling molecules such as MAPKKK17 (Menges et al., 2008), MAPKKK3 (Zipfel et al., 2004), and PP2C were found to be up-regulated (Grewal et al., 2012). Wen et al. (2002) also conducted an expression profile of 12 defense response genes where they observed constitutive expression of these genes but significantly induced under the influence of *Xoo* and the fungal pathogen causing blast disease, *Pyricularia grisea*. The RNA-Seq analysis of resistant (CBB23; a rice line carrying *Xa23* gene) and a susceptible (JG30) rice gentotypes led to identify several DEGs post infection by *Xoo* strain PXO99^A^. Moreover, several of the DEGs were up-regulated in CBB23 were related to immune responses like peroxidase, phytosulfokinase, RLKs, serine/threonine kinase, TFs (WRKY, NAC, MYB, bZIP, AP2/ERF etc.) and phytohormones (SA and ET) (Tariq et al., 2018). In another RNA-Seq analysis, where CBB23 was challenged with PXO99^A^ and its mutant P99M2 exploited 1235 DEGs at defferent time point (Wang et al., 2019). Publicly available *Xoo* infected rice microarray data analysis revealed the importance of mitochondria and chloroplast as an arena for up-regulated and down-regulated genes in response to *Xoo* infection (Kong et al., 2020). Therefore, the genes up-regulated in resistance varieties can be targeted for improvement through molecular breeding and transgenic approaches, and the rice genes that are up-regulated in susceptibility reaction against *Xoo* could be potential targets for silencing or genome editing.

### Proteomics Analysis of Products Encoded by Resistance Genes

Completion of sequencing of rice and *Xoo* genomes are significant accomplishments in host-pathogen interaction study. Even though genomes can evolve rapidly because of either transposable elements movement or from epigenetic changes, they are in general contemplated as highly static compared to their extremely dynamic proteomes. Therefore, there has been increased focus on elucidating functional aspect of proteins involved in rice-*Xoo* interaction. Comparative proteomics is emerging as a promising approach to develop a global understanding of protein expression under various conditions including attack by pathogen on the plant host (Agrawal and Rakwal, 2011; Ding et al., 2012). Using proteomic approaches, different biological processes including protein–protein interaction, post-translational modification, protein expression, etc. could be successfully analyzed during plant development, particularly during stress conditions (Hashiguchi et al., 2010). The induction of PR5 protein post nitrogen application was reported in case of *M. grisea* infection (Konishi et al., 2001). Stress related proteins such as superoxide dismutase (SOD), heat shock proteins (HSP), and dehydrins are induced in plant post rice yellow mottle virus (RYMV) inoculation (Ventelon-Debout et al., 2004). Proteomic study of sub-cellular organelles has been performed such as plasma membrane, vacuolar membrane, mitochondria, and chloroplast. Cytosolic and membrane protein study revealed the activation of proteins related to defense such as thaumatin-like protein (TLP) (PR5), probenazole-inducible protein 1 (PBZ10), (SOD), and peroxiredoxin (Mahmood et al., 2006). Transgenic rice lines overexpressing a TLP showed moderate level of resistance. After proteomic analysis, 440 protein spots were detected, where 10 proteins, including TLPs, were differentially expressed (five up-regulated and five down-regulated). TLP, ATP synthase B chain, glycine cleavage H protein, and 2-Cys peroxiredoxin were significantly up-regulated, whereas glycerol aldehyde dehydrogenase, salt induced protein, transketolase, and oxygen evolving enhancer protein 2 were down-regulated. Out of 10 differentially regulated protein spots, two did not show a substantial match with any known proteins (Mahmood et al., 2006). A study on plasma membrane (PM) proteome in rice revealed the involvement of at least eight PM-associated proteins in BB defense out of 20 protein spots induced after *Xoo* infection (Chen et al., 2007). These proteins were H^+^-ATPase, prohibitin (OsPHB2), hypersensitive-induced response protein (OsHIR1), quinone reductase, zinc finger, universal stress protein (USP), C2 domain protein, HSP, and protein phosphatase. A stable somatic hybrid line SH76 was developed using wild rice *O. meyeriana* and japonica rice cultivar (8411), which proteomic analysis revealed differential expression of 77 proteins including stress related proteins such as putative glutathione S- transferase, ascorbate peroxidase, and mitochondrial chaperonin-60 (Yu et al., 2008). Interestingly, differential induction stress associated proteins have been reported upon *Xoo* challenge, suggesting the likelihood of participation of mutual stress pathways. Some of the important candidate proteins activated in *O. longistaminata* post BB infection include cyclin-dependent kinase C, germin-like protein, putative r40c1, glutathione-dependent dehydroascorbate reductase 1 (GSH-DHAR1), and Ent-isokaur-15-ene synthase (Kumar et al., 2015).

### Metabolomics Analysis of Compounds From Resistant Plants

Cell signaling is the first molecular event that occurs during pathogen infection. Plant produces volatile metabolites/hormones such as ethylene, methyl jasmonate, methyl salicylate and nitric oxide as key mediators of host response to pathogen/pest infection and for systemic acquired resistance (Heuberger et al., 2014). These hormones conjugates with other metabolites like jasmonate and isoleucine to provide immunity (Staswick and Tiryaki, 2004). During plant-pathogen interaction, many small size molecules of different class such as homoserine, asparagine, and sphingolipids mediate signaling (Heuberger et al., 2014) and regulated through cross-talk between hormones like ethylene-jasmonate, nitric oxide, and jasmonate (Lorenzo et al., 2003; Jian and Wu, 2005).

Metabolism impacts cellular physiology and plays an essential role in biology. Recent advances in metabolomic analytical tool provides opportunity to dissect the layers of plant metabolic regulation, thus allowing bridging the gap between genome and the phenome (Metallo and Vander-Heiden, 2013). It also aids in identifying signature metabolites linked to agronomic traits, thereby plays dynamic role in crop improvement (Kumar et al., 2017; Sharma et al., 2018). Like other omics approach, metabolomics has the ability to examine the global expression of small metabolites that are involved in signaling, and morphological, physio-chemical responses produced during plant-pathogen interaction. In the past few years, researchers have put an effort to address the diverse mechanisms that rice plants use to adapt its metabolism during infection by *Xoo* and also discuss how metabolic flux alteration can be used to identify central regulatory nodes under pathological cell physiology. A resistant rice genotype responds to BB pathogenic invasion by increasing carotenoids, total phenolic, and flavonoid contents (Kumar et al., 2013). Boosted levels of flavonoids (cyanidin 3-galactoside, cyanidin 3-glucoside, delphinidin 3-arabinoside, and delphinidin 3-galactoside) in black scented rice protects it from diseases and these compounds are also known to have health promoting effects on human (Asem et al., 2015).

A high resolution metabolite QTL (mQTL) analysis of rice recombinant inbred line (RIL) population revealed ~ 2,800 mQTL associated with 900 metabolites (Gong et al., 2013). A major mQTL for aromatically acylated ﬂavonoids co-located within a 0.5-Mb region on chromosome 10, which consists of one acyltransferase gene *OsAT1* conferring BB resistance in rice by regulating lysophosphatidylcholines, has been identified (Mori et al., 2007; Gong et al., 2013). Leaf samples are excellent source to study the resistance mechanism in rice. Hence, evaluation of susceptible versus resistant mutants, varieties, or genotypes is an ultimate tool to interpret resistance mechanisms and identify defense related metabolites. The leaf metabolome was examined to identify the metabolites that might be responsible for differences between BB susceptible, wild type cultivar, and resistant transgenic rice plant (http://www.agilent.com/cs/library/applications/5989-6234EN.pdf). Studies suggest distinct subsets of metabolites at pre and post-invasion might coordinate during BB infection. For example, 42 metabolites could be predicted to be associated with BB resistance, 22 metabolites were connected to infection response, 25 metabolites could be formed by bacteria or in response to it, and a total of 170 metabolites were identified, which differentially expressed between the two-contrast line (Fisher and Sana, 2007). Recently, seed metabolome of a BB resistant line, C418/Xa23, which was generated through marker assisted breeding, and a transgenic variety C418-Xa21 were compared with the wild type susceptible progenitor (C418) (Wu et al., 2012). The study revealed distinct metabolite pattern in the seed of resistant line with significant decline in few common metabolites: amino acids (alanine, glycine, and tyrosine), organic acids (ferulic acid, succinic acid, and malic acid), and glycerol. Additionally, linoleic acid emerged as specific signature metabolite in the seed of resistant breeding line. Possibly, these metabolites regulate the Kreb’s cycle and amine biosynthesis, which drive the metabolic state and cell physiology (**Figure 3B**). There is a possibility to use these metabolites as novel discriminatory metabolites to identify BB resistant rice genotypes.

### System Biology Approach to Understand Rice-Xoo Interaction For Developing Strategies For Durable Resistance

Consolidating findings emanating from multiple omics platforms contributes to an improved understanding of metabolic pathways, genes, and gene-interaction networks responsible for the phenotypic changes that accompany plant-microbes interactions. A growing body of literature that integrates metabolomics, transcriptomics, and proteomics analyses suggests that significant metabolic alterations happen during plant-pathogen interactions (Wan et al., 2002; Oksman-Caldentey and Saito, 2005; Hollywood et al., 2006; Figueiredo et al., 2008; Tan et al., 2009; Choi et al., 2010; Sana et al., 2010; Paternain and Campion, 2013). However, similar approach for studying BB resistance in rice is presently lacking. To the best of our knowledge, only one study is conducted in rice to establish correlations between the metabolome and transcriptome (Sana et al., 2010). Examination of the metabolic profile of a resistant rice variety infected with *Xoo* suggested significant up-regulation of compounds such as pigments, rutin, fatty acids, and lipids in the resistant plant. The study compared the differential expression of genes in relation to these metabolite products and the corresponding enzymes, and their regulatory pathways. For instance, the transcriptomics and metabolomics data revealed strong correlation between decreased in glutamate levels with increased expression of *glutamate decarboxylase*, which encodes for an enzyme that catalyzes decarboxylation of glutamate to GABA in the *Xoo* challenged plants. Similarly, the increased expression of *Phenylalanine ammonia lyase* (gene product regulates phenyl propanoid pathway) was in coordination with the elevated level of phenylalanine in the *Xoo* challenged resistant plant. However, the expression of *isocitrate lyase*, *b-1,3-glucanase* and *chitinase* showed negative correlation with the metabolite data in the resistant plant as these genes are involved in degradation of fungal cell wall and provide resistance against fungal pathogen (O’Toole et al., 2001). We anticipate a remarkable increase in the studies that combine different omics platforms to provide better insights into BB resistance in rice.

## Epigenetics: A New Way to Improve Trait Understanding and Manipulation

To evaluate the epigenetic control responsible for resistance against *Xoo*, methylated regions of rice genome were using methylation sensitive amplified polymorphism (MSAP) technique, and cytosine methylation was screened by the bisulfite mapping technique (Akimoto et al., 2007). The rice seed were treated with 5-azadeoxycytidine (inhibitor of DNA methylation), and the progeny were grown in field. Among 1000 seeds treated with 5-azadeoxycytidine, only 35 seedlings survived and, out of that, two showed dwarf phenotypes. In contrast to susceptible wild type, line-2 showed constitutive expression of *Xa21G* and resistance against BB (Akimoto et al., 2007). Normally, the promoter remained at hyper-methylated condition, which silences *Xa21G* gene to cause susceptibility to *Xoo*. Besides DNA methylation, epigenetic regulatory pathways also regulate the initial step of plant-bacteria interaction through small RNAs (sRNAs), such as small interfering RNAs (siRNAs) and micro RNAs (miRNAs) (Carvalho et al., 2016). In *Arabidopsis* and legumes, miR393 is induced during pathogenic infection and confers resistance to associative and endophytic diazotrophic bacteria (AEDB) by attenuation of auxin signaling pathways (Navarro et al., 2006; Subramanian et al., 2008). Similarly, enhanced expression of miR160 in *Arabidopsis* during bacterial infection indicates its possible involvement in defense response (Fahlgren et al., 2007). In contrast, the down-regulation of miR160 and miR393 in maize caused suppression of defense response (Thiebaut et al., 2014). Genome editing tools such as ZFNs and TALEs were used for targeted epigenetic modifications in plants (Gaj et al., 2013). Presently, the CRISPR/Cas9 technology has become the widely accepted genome editing tool for plant modification including epigenetic modification (Malzahn et al., 2017; Van de Wiel et al., 2017). In mammalian system, targeted enhanced CpG methylation has been successfully demonstrated by using CRISPR/Cas9-DNMT3A as epigenetic modifier (Vojta et al., 2016). In future, such epigenetic modifications are expected to be routinely deployed for targeted methylation for disease resistance.

## Impact of Climate On BB Resistance Genes

The classical concept of Plant Pathology describes a plant disease as the result of interaction of host, pathogen and environment, commonly referred to as ‘disease triangle’. Thus, variations in any parameters of environment can remarkably affect the disease consequence. Several studies have shown that the effectiveness of ‘R’-gene mediated defense response can be substantially influenced by temperature variation (de Jong et al., 2000; Uauy et al., 2005; Webb et al., 2010; Zhu et al., 2010; Zhao et al., 2016). Webb et al. (2010) reported that near isogenic line IRBB4 owning the BB resistance gene *Xa4* exhibited much longer lesion at higher temperature regime (35:27°C, day:night) than in lower temperature regimes (29:21°C, day:night). Similarly, Dossa et al. (2017) observed that drought stress significantly reduced *Xa4* mediated BB resistance in rice. On the contrary, efficacy of BB resistance gene, *Xa7* was significantly improved at higher temperature (35:31°C, day:night) compared to lower temperature (29:21°C, day:night) in limiting BB severity and *Xoo* population (Webb et al., 2010). In a detailed study, Cohen et al. (2017) found that the level of *Xa7*-mediated BB resistance was comparatively stronger and faster at higher temperature as compared to lower temperature regime. These contrasting scenarios raise concerns about durability of single R gene and different gene combinations in the scenario of a rapidly changing climate. Recently, Dossa et al. (2020) demonstrated that near isogenic line IRBB 67 (owning BB resistance genes *Xa4* and *Xa7*) did not show any difference in lesion length at both lower and higher temperature regimes, indicating that the reduced effectiveness of *Xa4* at higher temperature did not affect the resistance level of IRBB 67.

## Perspective and Conclusions

The research achievements in the recent past have contributed to improved understanding of resistance mechanisms in rice against *Xoo* infection and also the pathways associated with susceptibility. Deploying multiple sets of carefully selected R-genes as gene-pyramids holds promise for developing improved rice cultivars/hybrids with durable and broad-spectrum BB resistance. Identification and utilization of new resistant genes/alleles, tapping the genetic variability in diverse germplasm, and generation novel variation in existing genes through gene-editing will be crucial to achieve sustained production of rice for ever-increasing population. Mapping and characterization of different BB R-genes have made marker-assisted selection a valuable tool to develop durable BB disease resistance in rice. Moreover, strategies for gene discovery based on genomics and proteomics together with transgene validation through genetic transformation are increasingly helping us understand the functional profiles of candidate genes. Research on this aspect has been immensely benefited from the increasing information on structure and function of major BB resistance genes. Although a smaller number of major R-genes have been cloned and characterized as of now, RGAs and DNA markers linked to resistance trait have been routinely deployed in BB resistant genotypes breeding. The possibility to surveil genomic variations and the evolution of virulence-avirulence factors have expanded further with the availability of complete genome sequences of rice and *Xoo*. Domestication of rice has indeed narrowed the genetic diversity particularly with respect to diverse allelic forms of resistance genes that may exist in wild relatives of *Oryza*. Hence, it becomes imperative to conduct large-scale survey of wild rice species to characterize novel BB resistance genes and also novel alleles of known resistance genes so that they can be gainfully used for rice improvements. In our opinion, the need of the hour is to make best possible use of information and resources available not only in rice but also in other related crop species so as to achive durable resistance against BB disease. For example, investigations on how wheat crop avoids infection by *Xoo* or how rice avoids infection by the wheat rust pathogen, *Puccinia gramins fsp. Tritici* may offer clues for developing broad spectrum resistance in rice against multiple pathogens including *Xoo*. Interestingly, the focus of the research is now making a paradigm shift from individual genes to the whole plant systems. We anticipate that a better-coordinated inter-disciplinary research may reduce redundancy and competition in specific area along with dedicating greater attention to previously unexplored research areas.

## Author Contributions

AK, RK, DS, SD, and AB designed the article. AK, RK, DS, SD, PS, and HS wrote the article. MP, NS, IG, GL, and RS corrected and improved the article. All authors contributed to the article and approved the submitted version.

## Funding

AK sincerely thanks University Grants Commission (UGC) start-up grant (no. F.30-392/2017 (BSR) and Madhya Pradesh Council of Science and Technology (Endt. no. 3879/CST/R&D/BioSci/2018) for the funding to the laboratory. RK thanks Science and Engineering Research Board (SERB), Department of Science and Technology (DST) for extending financial support (project #PDF/2016/002758). RS, GL, and PS thank the Indian Council of Agricultural Research, Department of Biotechnology, Department of Science and Technology and Council for Scientific and Industrial Research, Government of India for the generous funding and infrastructural support. SD sincerely thanks University Grants Commission (UGC) for start-up grant (no. F.30-420/2018 (BSR).

## Conflict of Interest

The authors declare that the research was conducted in the absence of any commercial or financial relationships that could be construed as a potential conflict of interest.
